# Scleral suture fixation of a foldable intraocular lens without a scleral flap or groove

**DOI:** 10.22336/rjo.2026.37

**Published:** 2026

**Authors:** Hasan Burhanettin Kaptı, Kader Kasar

**Affiliations:** 1 Department of Ophthalmology, Ordu University, Faculty of Medicine, Ordu, Turkey

**Keywords:** aphakia, scleral fixation, intraocular lens, suture techniques, BCVA = Best-corrected visual acuity, IOL = Intraocular lens, PCIOL = Posterior chamber intraocular lens, ACIOL = Anterior chamber intraocular lens, IFIOL = Iris fixated intraocular lens, IOP = Intraocular pressure, CME = Cystoid macular edema, SFIOL = Scleral-fixated intraocular lens, logMAR = Logarithm of the minimum angle of resolution, AS-OCT = Anterior segment optical coherence tomography, SD = Standard deviation

## Abstract

**Objective:**

This study aims to assess the surgical and visual outcomes of a sutured intraocular lens (IOL) fixation approach performed without creating a scleral flap or tunnel in aphakic eyes without posterior capsule support.

**Methods:**

In this retrospective study, we evaluated aphakic patients who underwent surgery using the ab externo scleral fixation technique that involved tying IOL fixation sutures on the sclera and placing the suture ends under the Tenon’s capsule without opening the scleral flap. Preoperative and postoperative visual acuity, refraction values, intraocular lens centralization, and operation-related complications were analyzed.

**Results:**

In the study involving 38 patients with an average follow-up period of 8.2 months, the best-corrected visual acuity (BCVA) improved from an average of 1.00 logMAR to 0.10 logMAR. Spherical equivalent refraction decreased from +10.03 D to +0.96 D. Intraocular lens decentration was observed in 7.9% of cases. No intraoperative complications were observed, while 5.2% of patients experienced postoperative corneal edema and 2.6% experienced macular edema.

**Discussion:**

The results of this study demonstrate that the ab externo scleral fixation technique without a scleral flap or groove is a safe and effective approach for secondary IOL implantation in aphakic eyes. The significant improvement in BCVA (from 1.00 to 0.10 logMAR) and the substantial reduction in spherical equivalent refraction confirm the technique’s optical efficacy. The low rates of intraoperative and postoperative complications, along with satisfactory IOL centration in 92% of cases, support the safety profile of this method. By positioning the suture ends beneath Tenon’s capsule rather than creating a scleral flap or tunnel, this technique reduces operative complexity. It minimizes the risk of suture exposure and conjunctival erosion, while maintaining reliable IOL fixation.

**Conclusions:**

Due to its short surgical length, low complication rate, and satisfactory visual results, this simplified sutured IOL fixing technique without a scleral flap or tunnel is safe and effective. Suture exposure is reduced, and the procedure is less invasive, with the suture ends under Tenon’s capsule. Larger, comparable, and prospective research is needed to assess the method’s long-term effects.

## Introduction

In posterior capsule insufficiency or zonular weakness, a secondary intraocular lens (IOL) is needed. Trauma, Marfan syndrome, pseudoexfoliation syndrome, congenital aniridia, and cataract surgical difficulties can cause such disorders. As the global population ages, the number of cataract procedures is increasing, leading to more secondary IOL implantation [1].

Recently, numerous secondary IOL fixation techniques have been developed. These include anterior chamber IOL (ACIOL), iris-fixated IOL (IFIOL), and scleral fixation IOL (SFIOL), each with its own specific advantages and risks [2-5]. ACIOL is rarely used in clinical practice because it can lead to anterior chamber angle injury, corneal endothelial injury, and even irreversible corneal endothelial decompensation [6]. IFIOLs present greater technical challenges compared to ACIOLs and are linked to a heightened risk of pupillary anatomical disruption, uveitis from pigment release, IOL dislocation, and vitreous hemorrhage [3]. Three types of SFIOLs are identified: sutured SFIOLs, glued SFIOLs, and sutureless SFIOLs. Glued SFIOLs effectively mitigate potential complications associated with sutures by anchoring the IOL ring beneath a scleral flap or within a scleral tunnel utilizing fibrin adhesive. The high cost of fibrin adhesive and the risk of prion-related infection post-surgery restrict the use of this procedure [7,8]. Sutureless techniques for scleral fixation have garnered attention as a solution to suture-related complications. These techniques are preferred for their procedural simplicity, potentially reduced surgical durations, and strong IOL fixation capability. Nonetheless, these methods pose specific challenges, including a considerable learning curve, potential intraoperative complications, and a risk of IOL dislocation [9,10]. The sutured SFIOL approach involves securing the intraocular lens to the sclera with 10-0 polypropylene, 9-0 polypropylene, or expanded polytetrafluoroethylene sutures. Sutured IOL fixation may result in problems, including suture rupture and suture erosion [11-13]. Nonetheless, with the ongoing advancement of science and technology, the approach to this operation is continually being streamlined, resulting in a lower rate of damage; thus, numerous clinical academics favor this surgical technique.

This research aims to assess the short-term anatomical and visual results of a modified surgical approach for IOL implantation using the transscleral suture-mediated IOL fixation technique. This method entails placing the suture ends beneath the Tenon’s capsule after tying a knot on the sclera, offering an alternative solution to specific complications associated with conventional sutured techniques. The objective is to mitigate issues commonly documented in the literature, including suture exposure and conjunctival irritation. The clinical efficacy and safety of the technique were retrospectively analyzed using parameters such as surgical efficacy, complication rates, visual gain, and IOL stability.

## Methods

This retrospective case series, conducted between January 2016 and March 2024, involved consecutive aphakic eyes that underwent posterior chamber intraocular lens (PCIOL) implantation using a modified transscleral fixation technique, which involved placing the suture knot on the sclera and threading its ends under the Tenon’s capsule without creating a scleral flap or tunnel. The study was conducted in accordance with the Declaration of Helsinki and was approved by the institutional ethics committee (Approval No. 2025/379). All patients signed an informed consent form before surgery.

Inclusion criteria included aphakia resulting from complex cataract surgery, trauma, or lens subluxation due to inadequate capsular support; participants aged 18 years and older; and a minimum postoperative follow-up period of 3 months. Exclusion criteria included retinal pathologies affecting vision (retinal detachment, diabetic retinopathy, macular degeneration, etc.), previous glaucoma, vitreoretinal or corneal surgery, uncontrolled glaucoma, previous unsuccessful scleral fixation surgery, and missing operative and postoperative data.

**Fig. 1:**
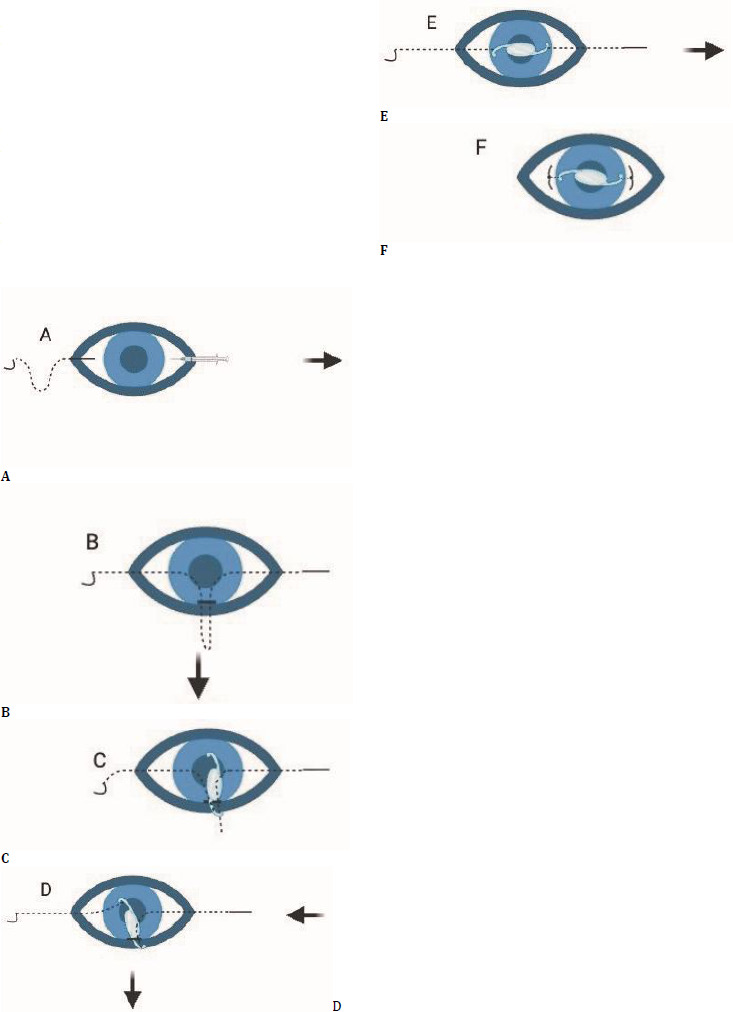
Surgical Steps of Scleral Fixation of a Foldable Intraocular Lens Using a Suture Technique: (A) Transscleral passage of a double-armed 10-0 polypropylene suture and a 27-G needle. (B) Suture retrieval through the opposite scleral entry and externalization via corneal incision. (C) IOL insertion; one haptic is tied to the suture. (D) IOL rotation and fixation of the second haptic. (E) Centering of the IOL. (F) Suture is anchored to the sclera, and its ends are left long

All surgeries were performed by the same surgeon under retrobulbar anesthesia. Conjunctival peritomy was created at 180° intervals at 3 and 9 o’clock positions, followed by hemostasis with a cautery. In the nasal and upper temporal quadrants, side port incisions were made at 120° intervals. A clear corneal incision measuring 2.75 mm was made in the upper quadrant. Anterior vitrectomy was performed when vitreous was detected in the anterior chamber. The anterior chamber was filled with 1.6% hyaluronic acid-4.0% chondroitin sulfate (Discovisc, Alcon Laboratories, Inc., Fort Worth, TX, ABD). A double-needle 10-0 polypropylene suture (PC-9, Ethicon Inc.; Somerville, NJ, USA) was passed transsclerally ab externo 2 mm behind the limbus. The suture was removed from the opposite quadrant using a 26-gauge needle passed 2 mm behind the limbus. The suture was subsequently removed from the anterior chamber with microforceps. A foldable IOL (SAF 6125SQ, Optima Lens, Excellent Hicare Pvt Ltd, India) was implanted in the anterior chamber via a corneal incision. One of the haptics was left outside. The part of the suture brought outside was cut, passed through the hole in the external haptic, and tightly tied, and the haptic was introduced into the anterior chamber. The procedure was repeated for the second haptic implant. Following confirmation of the IOL’s centration behind the iris, the ends of the 10-0 polypropylene sutures were secured to the sclera. Both suture ends were intentionally left approximately 5-6 mm in length. The long suture ends following subtenon dissection were positioned beneath the tenon. This method sought to minimize the risk of exposure, conjunctival erosion, and discomfort at the knot site. The conjunctiva was sutured with 8/0 Vicryl (Polyglactin 910, Ethicon, Somerville, NJ, USA). All incisions were moistened. In the event of leakage at the main corneal incision, it was sutured using a 10-0 Prolene suture (CIF-4; Ethicon, Somerville, NJ, USA) (Fig. 1).

The demographic data for all patients were recorded. In pre- and post-operative examinations, best-corrected visual acuity (BCVA, LogMAR), refractive outcomes expressed as spherical equivalents, corneal astigmatism, and intraocular pressure (IOP) were assessed. Additionally, we recorded surgical duration, the centralization of the IOL assessed via slit lamp biomicroscopy, and information regarding intraoperative and postoperative complications, the need for additional surgical intervention, and the follow-up duration. The measurement of spherical equivalent and corneal astigmatism was performed using an autorefractometer-keratometer (Topcon Auto Refractometer-Keratometer, Tokyo, Japan), and intraocular pressure was measured using a non-contact tonometer (Auto Tonometer TX-F; Topcon, Japan).

Statistical analyses were performed using IBM SPSS Statistics software (version 22.0, IBM Corp., Armonk, NY, USA). The normality of the data distribution was assessed using the Shapiro-Wilk test. The paired samples t-test was employed to compare preoperative and postoperative parameters with a normal distribution. In contrast, the Wilcoxon signed-rank test was used for data that were not normally distributed. Categorical variables were analyzed using the chi-square test or Fisher’s exact test, as appropriate. *P* values < 0.05 were considered statistically significant.

## Results

Thirty-eight eyes in 32 patients who underwent secondary IOL implantation were evaluated. The mean age of the patients was 67.2 ± 10.4 years; 23 were male (60.5%), and 15 were female (39.5%). In most cases, the cause of aphakia was complicated cataract surgery (32 eyes, 84.2%). The other cause of aphakia was IOL dislocation (6 eyes, 15.8%). Demographic data are shown in Table 1.

**Table 1:** Demographic and Clinical Characteristics

Variable	Value
Patients / Eyes	32 / 38
Age (years) mean ± SD range	67.2 ± 8.3 50-89
Sex (%) Male Female	25 (65.8) 13 (34.2)
Right / Left eyes	23 / 15
Surgical time (min) mean ± SD range	44.5 ± 7.4 32-60
Follow-up duration (months) mean ± SD range	8.2 ± 2.3 3-12

* SD, standard deviation

The best-corrected visual acuity (BCVA) before surgery was 1.00 ± 0.12 logMAR on average, while it reached 0.10 ± 0.07 logMAR in the postoperative period with a significant increase (*p* < 0.001). The mean spherical equivalent value was +10.03 ± 4.6 D preoperatively and +0.96 ± 1.4 D postoperatively, and this difference was statistically significant (*p* < 0.001).

Postoperative corneal astigmatism values increased from a preoperative 1.22 ± 0.88 D to 1.52 ± 0.98 D, and the increase was not statistically significant (*p* = 0.51). Intraocular pressure (IOP) was measured at 14.2 ± 2.8 mmHg in the preoperative period and 14.7 ± 3.1 mmHg in the postoperative period; the difference was not statistically significant (*p* = 0.53).

The postoperative biomicroscopic evaluation indicated that the intraocular lens placement was 92% central. Decentration was observed in three eyes (7.9%), yet none necessitated revision surgery. The average follow-up period was 8.2 ± 2.5 months.

**Fig. 2:**
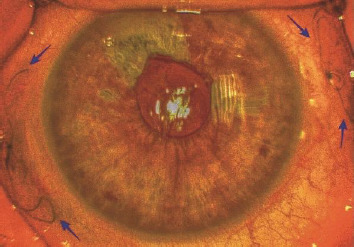
Postoperative image showing sutures under the tenon (blue arrows)

No intraoperative complications were noted. During the postoperative period, transient corneal edema was observed in 2 cases (5.2%), while cystoid macular edema occurred in 1 case (2.6%). Corneal edema was resolved through medical intervention. Macular edema resolved following a single intravitreal injection of triamcinolone. In two patients (5.2%), suture exposure occurred within the first month post-surgery. These patients required reoperation to open the conjunctiva, reposition the suture, place it beneath the Tenon’s capsule, and subsequently close the conjunctiva again. Additional surgical intervention was not required for any other patients (Fig. 2).

## Discussion

In this study, the surgical and visual outcomes of IOL fixation using the ab externo suture technique, without creating a scleral flap, in aphakic eyes were evaluated. These findings indicated that the sutured ab externo approach without a flap might be a safe and effective alternative. This technique reduces the risk of suture erosion by placing the suture beneath Tenon’s capsule without requiring a scleral flap, thereby simplifying the surgical process.

In this study, BCVA showed a significant improvement, changing from an average of 1.00 logMAR to 0.10 logMAR. Additionally, spherical equivalent refraction decreased from +10.03 D to +0.96 D. Comparative studies in the literature have reported comparable visual gains with Yamane and similar sutureless techniques [5,14]. Zhang et al.’s meta-analysis revealed no significant difference in BCVA between the sutured and Yamane techniques. Zhang’s study indicated that sutured techniques typically required a longer surgical duration but demonstrated comparable outcomes for refractive error [1].

Investigations assessing both sutured and sutureless scleral fixation techniques have indicated dislocation rates varying from 0 to 15 percent [15-17]. Our investigation revealed that the IOL decentration rate was 7.9%, consistent with existing literature. The investigation conducted by Kim et al. compared the modified double-flanged sutureless technique with the traditional sutured method. Findings indicated that the double-flanged group exhibited lower myopic deviation, better centering, and fewer iris captures [18]. This situation highlighted several benefits of sutureless techniques, while our sutured-yet-flapless method may also be competitive in terms of stability. On the other hand, meta-analysis studies examining sutureless techniques emphasize the risk of IOL tilt or haptic protrusion [19].

Notably, no intraoperative complications were observed in our study. In the postoperative period, only transient corneal edema (5.2%) and cystoid macular edema (CME) (2.6%) were observed, and these rates are consistent with those reported in the literature. In a study comparing three scleral fixation techniques, the CME rate was 5.7% in the suture fixation group, and 4.8% and 7.4% in the sutureless groups, respectively [16]. Similarly, in another study comparing the sutured SFIOL technique with the sutureless Yamane technique, CME rates were reported as 13.0% and 1.9%, respectively [20]. Another important complication encountered after SFIOL surgery is postoperative corneal edema. There are studies in the literature reporting this complication at quite high rates of 35.7% and 18.9% [21,22]. The low rates of these complications in our study support the safety of the technique used.

Malbran first described transscleral suture-assisted IOL fixation in 1986, and due to its low postoperative complication rate, it has become a relatively common surgical approach [23]. Previous studies have identified suture-related complications associated with this approach, including inflammation, suture erosion, and suture disruption. Approaches such as turning the suture knots toward the eye and covering the suture ends with scleral pockets or flaps have been used to prevent these complications [19]. However, creating a scleral flap can prolong the operation time and create technical difficulties. In the scleral fixation technique used in our study, after the IOL fixation sutures were knotted on the sclera, the suture ends were left long without being embedded in the sclera. They were laid under the Tenon’s capsule on the sclera. This method reduces the risk of exposure and conjunctival erosion while also eliminating the need for a scleral flap. In this respect, it is easier to apply than traditional sutured scleral fixation methods. One of the main concerns regarding the procedure we used in this study was that 10-0 polypropylene sutures might deteriorate over time, ultimately leading to IOL subluxation or dislocation. According to Lockington et al., the use of 10-0 polypropylene sutures resulted in suture breakage rates ranging from 1.3% to 27.9% after 3-8 years [24]. However, during our follow-up period, we did not encounter such a complication in our study.

Sutureless techniques, particularly the Yamane method, have gained popularity in recent years. However, these approaches require a significant learning curve and involve certain intraoperative technical challenges. Several studies have reported complications associated with these methods, including long-term stability issues, dislocation, and IOL tilt [14,25]. The technique employed in this study offers a reliable alternative for surgeons experienced with suturing, enabling secure IOL fixation.

This research presented several limitations. The retrospective design of the study, lacking a control group, might introduce potential selection and reporting biases. The absence of direct comparisons with alternative scleral fixation techniques, such as flanged sutureless or scleral flap methods, restricts the evaluation of the method’s relative efficacy. The average follow-up period of 8.2 months is insufficient for obtaining reliable long-term data on late complications, including suture loosening, breakage, or late intraocular lens decentration. Long-term and prospective studies would facilitate a more precise evaluation of the method’s durability and safety. The small patient cohort limits statistical power and complicates the detection of rare complications. Conducting all surgeries by a single experienced surgeon enhances surgical standardization but limits the generalizability of outcomes across surgeons. The assessment of centralization and tilt of the intraocular lens was conducted subjectively via slit lamp biomicroscopy. Using objective techniques, such as anterior segment optical coherence tomography (AS-OCT) or ultrasonic biomicroscopy, might have yielded more accurate results. The study assessed only visual acuity and refractive outcomes, omitting analysis of functional parameters such as contrast sensitivity, patient satisfaction, and quality of life. This study, despite its limitations, represents one of the few series that provide clinical data on a technique performed without forming a scleral flap and with suture ends positioned beneath the Tenon’s capsule, thereby contributing to the existing literature.

## Conclusion

In this study, it was demonstrated that a simplified scleral fixation technique, based on placing the knot on the sclera and placing the suture ends under the Tenon’s capsule without creating a scleral flap in aphakic eyes, is an effective and safe method. Thanks to the applied technique, the surgical time was shortened, no intraoperative complications were observed, postoperative visual acuity increased significantly, and intraocular lenses were largely centrally positioned. The suture exposure rate was low, and the complications observed were minimal and reversible. This approach offers a practical alternative to existing methods, as it is less invasive, does not require creating a scleral flap or tunnel, and ensures knot burial with Tenon’s support. However, larger sample sizes, comparative, and long-term prospective studies are needed to evaluate the long-term efficacy and safety of this method.

## References

[1] Zhang C, Palka C, Zhu D, Lai D, Winokur J, Shwani T, DeAngelis MM, Reynolds AL (2024). Clinical Outcomes in Scleral Fixation Secondary Intraocular Lens with Yamane versus Suture Techniques: A Systematic Review and Meta-Analysis. J Clin Med.

[2] Chan TC, Lam JK, Jhanji V, Li EY (2015). Comparison of outcomes of primary anterior chamber versus secondary scleral-fixated intraocular lens implantation in complicated cataract surgeries. Am J Ophthalmol.

[3] Koc H, Ozer MA (2025). Iris Suture-Fixed Intraocular Lens Surgery as the Primary Preference in Aphakic Patients Without Capsular Support: Complications and Long-Term Results. Turkish Journal of Clinical and Experimental Ophthalmology.

[4] Kjeka O, Bohnstedt J, Meberg K, Seland JH (2008). Implantation of scleral-fixated posterior chamber intraocular lenses in adults. Acta Ophthalmol.

[5] Carlà MM, Boselli F, Giannuzzi F, Caporossi T, Gambini G, Mosca L, Savastano A, Rizzo S (2023). Sutureless scleral fixation Carlevale IOL: a review on the novel designed lens. Int Ophthalmol.

[6] Liu Z, Xie Q, Chen X, Xie B, Cai S (2023). Effect of sutureless scleral fixed intraocular lens implantation on aphakic eyes: a system review and meta-analysis. BMC Ophthalmol.

[7] Kang JJ, Ritterband DC, Tolees SS, Seedor JA (2015). Outcomes of glued foldable intraocular lens implantation in eyes with preexisting complications and combined surgical procedures. J Cataract Refract Surg.

[8] McKee Y, Price FW, Feng MT, Price MO (2014). Implementation of the posterior chamber intraocular lens intrascleral haptic fixation technique (glued intraocular lens) in a United States practice: Outcomes and insights. J Cataract Refract Surg.

[9] Czajka MP, Frajdenberg A, Stopa M, Pabin T, Johansson B, Jakobsson G (2020). Sutureless intrascleral fixation using different three-piece posterior chamber intraocular lenses: a literature review of surgical techniques in cases of insufficient capsular support and a retrospective multicentre study. Acta Ophthalmol.

[10] Wang T, Chen Y, Lu J, Li N, Min H (2023). A novel surgical approach for fixation of a posterior chamber intraocular lens of Rayner 620 H with Gore-Tex suture. BMC Ophthalmol.

[11] Junqueira NB, Chaves LJ, Poli-Neto O, Scott IU, Jorge R (2021). Scleral fixation using a hydrophilic four-haptic lens and polytetrafluoroethylene suture. Sci Rep.

[12] Bechay J, Rosenberg S, Flynn E, Bitar M (2024). Unburied polytetrafluoroethylene scleral suture erosions and failure of pericardial graft revision. Am J Ophthalmol Case Rep.

[13] Napolitano P, Filippelli M, Carosielli M, Costagliola C, Dell'Omo R (2023). Scleral flaps, pars plana vitrectomy and gore-tex sutured posterior chamber intraocular lens placement: a case series and review of literature. Front Ophthalmol (Lausanne).

[14] Yamane S, Sato S, Maruyama-Inoue M, Kadonosono K (2017). Flanged Intrascleral Intraocular Lens Fixation with Double-Needle Technique. Ophthalmology.

[15] Patel LG, Starr MR, Ammar MJ, Yonekawa Y (2020). Scleral fixated secondary intraocular lenses: a review of recent literature. Curr Opin Ophthalmol.

[16] Boz AAE, Atum M, Özmen S, Yuvacı İ Çelik E (2023). Comparison of three different intraocular lens implantation techniques in the absence of capsular support: sutured scleral, haptic flanged intrascleral, and four flanged intrascleral fixations. Int Ophthalmol.

[17] Shelke K, Rishi E, Rishi P (2021). Surgical outcomes and complications of sutureless needle-guided intrascleral intraocular lens fixation combined with vitrectomy. Indian J Ophthalmol.

[18] Kim J, Lee PY, Park MS, Cho BJ, Kwon S (2024). Comparison of outcomes between modified double-flanged sutureless scleral fixation and conventional sutured scleral fixation. Sci Rep.

[19] Tripathi M, Rao S, Sinha R (2025). Scleral-fixated IOLs-A comprehensive review of current practices and emerging trends. Indian J Ophthalmol.

[20] Ishikawa H, Uchida K, Terasaki H, Sakamoto T, Kakinoki M, Ohji M, Jujo T, Takagi H, Mitamura Y, Yamada Y, Takamura Y, Sugimoto M, Kondo M, Yoshida S, Fukuyama H, Gomi F (2025). Cystoid macular oedema after flanged intraocular lens scleral fixation using the Yamane technique: a multicentre cohort study. Sci Rep.

[21] Mo B, Li SF (2020). Novel use of an adjustable single 8-0 polypropylene suture of scleral fixation without conjunctival dissection. BMC Ophthalmol.

[22] Leuzinger-Dias M, Lima-Fontes M, Rodrigues R, Oliveira-Ferreira C, Madeira C, Falcão-Reis F, Fernandes V, Rocha-Sousa A, Falcão M (2021). Scleral Fixation of Akreos AO60 Intraocular Lens Using Gore-Tex Suture: An Eye on Visual Outcomes and Postoperative Complications. J Ophthalmol.

[23] Malbran ES, Malbran E, Negri I (1986). Lens guide suture for transport and fixation in secondary IOL implantation after intracapsular extraction. Int Ophthalmol.

[24] Lockington D, Ali NQ, Al-Taie R, Patel DV, McGhee CN (2014). Outcomes of scleral-sutured conventional and aniridia intraocular lens implantation performed in a university hospital setting. J Cataract Refract Surg.

[25] Schranz M, Reumüller A, Kostolna K, Novotny C, Schartmüller D, Abela-Formanek C (2023). Refractive outcome and lens power calculation after intrascleral intraocular lens fixation: a comparison of three-piece and one-piece intrascleral fixation technique. Eye Vis (Lond).

